# Supplementary material for: Early elevated IFNα is a key mediator of HIV pathogenesis
Source: Commun Med (Lond). 2024 Mar 19;4:53. doi: 10.1038/s43856-024-00454-6 (PMC10951235; doi:10.1038/s43856-024-00454-6)
Supplement: Supplementary file 1 — Supplementary Information [file 43856_2024_454_MOESM1_ESM.pdf]

Supplementary Tables and Figures:

Supplementary Table 1: Clinical data for HIV patients

Table 1a : EC patients

**EC**	HIV dx	Age	Sex	Sample date	CD4 at sample date	VL	HLA B57	Race	HIV risk factor	Treatment at the time of sampling
3	1990-2000	60-80	M	10/12/2012	1140	334	-	AA	IDU	None
4	1990-2000	40-60	F	22/08/2011	952	<40	+	AA	HS	None
6	1990-2000	40-60	F	31/05/2011	1889	<75	+	AA	IDU	None
8	1980-1990	40-60	M	01/02/2010	1018	<40	+	AA	IDU	None
9	2000-2010	40-60	M	25/09/2014	496	<40	+	AA	IDU	None
11	1990-2000	60-80	M	10/72/2011	917	<40	+	AA	IDU	None
13	1990-2000	40-60	M	21/02/2012	864	<40	-	AA	IDU	None
31	1990-2000	40-60	M	23/05/2012	587	<48	-	AA	MSM	None
32	1990-2000	40-60	F	23/05/2012	643	<48	-	AA	IDU	None
42	1990-2000	60-80	M	13/11/2013	731	<220	+	AA	IDU	None
47	2000-2010	40-60	F	01/12/2013	891	<20	-	AA	IDU	None
51	2000-2010	40-60	F	20/10/2014	1250	<40	+	AA	HS	None
52	1990-2000	40-60	M	01/08/2012	340	<20	+	AA	IDU	None
55	1990-2000	40-60	M	01/02/2013	482	50	-	AA	IDU	None
58	1990-2000	40-60	F	01/03/2013	1745	59	-	AA	HS	None
63	1980-1990	40-60	F	24/01/2014	632	32	+	AA	IDU	None
65	1990-2000	40-60	M	01/06/2013	1792	<48	+	AA	HS	None
68	2000-2010	40-60	F	01/07/2010	584	169	+	AA	HS	None

Dx : Diagnosis; M : Male; F : Female; AA : Afro American ; IDU : Injection Drug Use ; HS : Homosexual
MSM : Men who have sex with men

Table 1b : non-EC patients

Untreated patient study_ID	Age	Sex	sample date	CD4 at sample date	VL	Treatment at the time of sampling	Origin
Non-EC 1	NA	NA	03/11/2004	506	23808	None	NIH (Bethesda)
Non-EC 2	NA	NA	22/04/2004	516	58059	None	
Non-EC 3	NA	NA	01/10/2005	230	153719	None	
Non-EC 4	NA	NA	27/09/2005	327	132773	None	
Non-EC 5	NA	NA	19/06/2006	419	1715	None	
Non-EC 6	NA	NA	04/10/2007	513	12015	None	
Non-EC 7	NA	NA	13/08/2007	447	21701	None	
Non-EC 8	NA	NA	13/03/2008	319	4785	None	
Non-EC 9	NA	NA	05/11/2011	756	7011	None	
Non-EC 10	NA	NA	08/10/2005	445	407	None	
Non-EC 11	NA	NA	11/10/2009	292	3012	None	
Non-EC 12	NA	NA	10/07/2010	477	29257	None	
Non-EC 13	NA	NA	07/22/08	639	<50	None	
Non-EC 14	NA	NA	11/17/2008	267	26059	None	
Non-EC 15	NA	NA	03/23/2006	470	5384	None	
Non-EC 16	NA	NA	11/07/2012	740	11417	None	
Non-EC 17	NA	NA	01/28/2014	344	63954	None	
Non-EC 18	NA	NA	11/04/2010	872	7180	None	
Non-EC 19	NA	NA	12/03/2002	196	17809	None	
Non-EC 20	20-40	F	10/26/2020	470	2480	None	Laboratoire de Référence SIDA (Liège)
Non-EC 21	20-40	M	12/03/2020	100	24800	None	
Non-EC 22	20-40	M	12/03/2020	139	32800	None	
Non-EC 23	20-40	F	12/03/2020	356	9790	None	
Non-EC 24	20-40	M	12/08/2020	390	588000	A few days	
Non-EC 25	20-40	M	12/09/2020	1064	14700	None	
Non-EC 26	20-40	M	12/14/2020	30	850000	None	
Non-EC 27	40-60	M	02/24/2021	20	824	A few days	
Non-EC 28	40-60	F	03/05/2021	280	82700	None	
Non-EC 29	20-40	M	03/16/2021	577	157000	None	
Non-EC 30	20-40	F	03/19/2021	180	73700	None	

NA : Non Available

Supplementary Table 2: mAb list

Table 2a: mAb list for the immune cells culture phenotype

Markers	Fluorochrome	Clone	Origin	Dilution
**CD3**	AF532	UCHT1	Invitrogen	1/20
**CD4**	BV510	RPA-T4	BioLegend	1/40
**CD8**	BV750	RPA-T8	BioLegend	1/300
**CD25**	BV786	M-A251	BD Biosciences	1/40
**CD38**	PerCPeF710	HB7	Invitrogen	1/40
**CD56**	BV711	HCD56	BioLegend	1/150
**CD16**	Ef450	eBioCB163	Invitrogen	1/600
**NKG2D**	PE	AD11	BD Biosciences	1/20
**CD95**	BV421	DX2	BD Biosciences	1/10
**7AAD**	7AAD		Sigma Aldrich	1/50

Table 2b: mAb list for the immune cells panel

	Markers	Fluorochrome	Clone	Origin	Dilution
**Immune cell types**	CD3	AF532	UCHT1	Invitrogen	1/20
	CD4	BV510	RPA-T4	BioLegend	1/40
	CD8	BV750	RPA-T8	BioLegend	1/300
	CD56	BV711	HCD56	BioLegend	1/150
	CD16	Ef450	eBioCB163	Invitrogen	1/600
	TCR-γδ	BV480	11F2	BD Biosciences	1/40
	CD19	BV750	HIB19	Biolegend	1/40
	CD14	AF647	MOP9	BD Biosciences	1/300
	CD123	PerCpCy5.5	7G3	BD Pharmingen	1/10
	CD11c	BV605	3.9	BioLegend	1/20
**Immune Activation / Maturation**	CD45RA	FITC	REA562	Miltenyi Biotech	1/400
	CCR7	BV421	G043H7	Biolegend	1/40
	CD28	APC-R700	CD28.2	BD Biosciences	1/80
	CD25	BV786	M-A251	BD Biosciences	1/40
	HLA-DR	APC-Cy7	1243	Biolegend	1/1000
	CD26	PE	BA5b	Biolegend	1/300
	CD39	PeCy7	A1	Biolegend	1/300
	CD38	PerCPeF710	HB7	Invitrogen	1/40
**Immune checkpoint**	PD1	BV650	EH12.2H7	Biolegend	1/40
	CTLA-4	PeCy5	BNI3	BD Biosciences	1/20
	KIR2DL1	APC	REA284	Miltenyi Biotech	1/100
	KIR3DL1/DL2	APC	REA970	Miltenyi Biotech	1/100
	KIR2DL2/DL3	APC	DX27	Miltenyi Biotech	1/20
	KIR2DL5	APC	REA955	Miltenyi Biotech	1/100
**T-cell function**	Foxp3	PeCF594	236A/E7	BD Biosciences	1/100
**Viability**	Zombie	NIR		Biolegend	1/300

Table 2c: mAb list for CD8^{+} T-cells panel

	Markers	Fluorochrome	Clone	Origin	Dilution
**Immune cell types**	CD3	AF532	UCHT1	Invitrogen	1/20
	CD4	BV510	RPA-T4	BioLegend	1/40
	CD8	BV570	RPA-T8	BioLegend	1/300
	CD56	APC-Cy7	HCD56	BioLegend	1/80
**Immune Activation / Maturation**	CD45RA	BV421	HI100	BioLegend	1/80
	CCR7	BV785	G043H7	BioLegend	1/20
	CD28	APC-R700	CD28 .2	BD Biosciences	1/80
	Hélios	PE-Dazzle594	22F6	BioLegend	1/40
**Immune checkpoint**	NKG2A	PE-Vio770	REA110	Miltenyi Biotech	1/400
	KIR2DL1	APC	REA284	Miltenyi Biotech	1/100
	KIR3DL1/DL2	APC	REA970	Miltenyi Biotech	1/100
	KIR2DL2/DL3	APC	DX27	Miltenyi Biotech	1/20
	KIR2DL4	APC	REA768	Miltenyi Biotech	1/100
	KIR2DL5	APC	REA955	Miltenyi Biotech	1/100
	NKG2C	VioBright	REA205	Miltenyi Biotech	1/100
	Nkp30	PE-Cy5	Z25	Beckman Coulter	1/20
	Nkp44	PE-Cy5	Z231	Beckman Coulter	1/20
	Nkp46	PE-Cy5	BAB281	Beckman Coulter	1/20
	PD1	BV650	EH12.2H7	BioLegend	1/40
**Viability**	zombie	NIR		BioLegend	1/300

Table 2d: mAb list for cytotoxic CD8^{+} T-cells panel

	**Markers**	**Fluorochrome**	**Clone**	**Origin**	**Dilution**
	CD3	AF532	UCHT1	Invitrogen	1/20
	CD4	BV510	RPA-T4	BioLegend	1/40
	CD8	BV570	RPA-T8	BioLegend	1/300
	CD56	APC-Cy7	HCD56	BioLegend	1/80
**CD8+ cytotoxic T-cells**	HLA1-a	BV711	pentamer	Proimmune	1/20
	HLA-E	PE	E*01 :01	Proimmune	1/20
**Immune Activation / Maturation**	CD45RA	BV421	HI100	BioLegend	1/80
	CCR7	BV785	G043H7	BioLegend	1/20
	CD28	APC-R700	CD28 .2	BD Biosciences	1/80
	Helios	PE-Dazzle594	22F6	BioLegend	1/40
	Nkp30	PE-Cy5	Z25	Beckman Coulter	1/20
	Nkp44	PE-Cy5	Z231	Beckman Coulter	1/20
	Nkp46	PE-Cy5	BAB281	Beckman Coulter	1/20
**Immune checkpoint**	NKG2A	PE-Vio770	REA110	Miltenyi Biotech	1/400
	KIR2DL1	APC	REA284	Miltenyi Biotech	1/100
	KIR3DL1/DL2	APC	REA970	Miltenyi Biotech	1/100
	KIR2DL2/DL3	APC	DX27	Miltenyi Biotech	1/20
	KIR2DL5	APC	REA955	Miltenyi Biotech	1/100
	NKG2C	VioBright	REA205	Miltenyi Biotech	1/100
	PD1	BV650	EH12.2H7	BioLegend	1/20
**Imune cell function**	GrzB/perf	PerCP-cy5.5	QA16A02/B-D48	Biolegend	1/100
**Viability**	zombie	NIR		BioLegend	1/300

Supplementary Table 3: IFN type I effects on immune cell subtypes and its correlation with suppressive cytokines.

A-Known potential pathologic effects during chronic viral infection of IFN type I

Cellular mechanism	Cell type	effects	References
**Homeostasis**	T-cells	Inhibits IL-7 signaling pathway	*(1,2)*
**Proliferation**	T-cells	Inhibits T-cell proliferation	*(3)*
**Hyper-activation**	T-cells	Increases of CD38, HLA-DR	*(4,5,6,7)*
**Loss of function**	T-cells	Enhances PD-1 expression	*(8,7,9,10)*
		Decreases CD25 expression	*(11)*
		Decreases CD28 expression	*(12)*
	DCs and other APCs	Upregulates PD-L1, IL-10	*(13,14)*
**Apoptosis**	T-cells	Enhances expression of CD95, Bak, TRAIL	*(15,16,17)*
	DCs and other APCs	Upregulates and pro-apoptotic molecules	*(18,19)*
**Involution**		Induces thymic involution	*(20,21)*

B-Known correlation between IFN type I and immune suppressive cytokines

Suppressive cytokine	effects	References
**TGF-β1**	IFNα enhances TGF-β1 signaling pathways	*(22)*
**IL-10**	IFNα promotes $CD4^+$T-cells differentiation into suppressive IL-10 secreting Tr1 cells	*(23,24)*

References:

1. Nguyen, T. P. *et al.* Interferon- *α* inhibits CD4 T cell responses to interleukin-7 and interleukin-2 and selectively interferes with Akt signaling. *J. Leukoc. Biol.* **97**, 1139–1146 (2015).

2. Cha, L., de Jong, E., French, M. A. & Fernandez, S. IFN-α Exerts Opposing Effects on Activation-Induced and IL-7–Induced Proliferation of T Cells That May Impair Homeostatic Maintenance of $CD4^+$ T Cell Numbers in Treated HIV Infection. *J. Immunol.* **193**, 2178–2186 (2014).

3. Dondi, E., Rogge, L., Lutfalla, G., Uzé, G. & Pellegrini, S. Down-modulation of responses to type I IFN upon T cell activation. *J. Immunol. Baltim. Md 1950* **170**, 749–756 (2003).

4. Boasso, A., Hardy, A. W., Anderson, S. A., Dolan, M. J. & Shearer, G. M. HIV-Induced Type I Interferon and Tryptophan Catabolism Drive T Cell Dysfunction Despite Phenotypic Activation. *PLoS ONE* **3**, e2961 (2008).
5. Guo, K. *et al.* Qualitative Differences Between the IFNα subtypes and IFNβ Influence Chronic Mucosal HIV-1 Pathogenesis. *PLOS Pathog.* **16**, e1008986 (2020).
6. Alter, G. *et al.* Sequential deregulation of NK cell subset distribution and function starting in acute HIV-1 infection. *Blood* **106**, 3366–3369 (2005).
7. Zhen, A. *et al.* Targeting type I interferon-mediated activation restores immune function in chronic HIV infection. *J. Clin. Invest.* **127**, 260–268 (2017).
8. Cheng, L. *et al.* Blocking type I interferon signaling enhances T cell recovery and reduces HIV-1 reservoirs. *J. Clin. Invest.* **127**, 269–279 (2016).
9. Terawaki, S. *et al.* IFN-α directly promotes programmed cell death-1 transcription and limits the duration of T cell-mediated immunity. *J. Immunol. Baltim. Md 1950* **186**, 2772–2779 (2011).
10. Hafler, D. *et al.* Type I Interferon Transcriptional Network Regulates Expression of Coinhibitory Receptors in Human T cells. *Res. Sq.* rs.3.rs-133494 (2021) doi:10.21203/rs.3.rs-133494/v1.
11. Zella, D. *et al.* IFN-alpha 2b reduces IL-2 production and IL-2 receptor function in primary CD4+ T cells. *J. Immunol. Baltim. Md 1950* **164**, 2296–2302 (2000).
12. Lanna, A. *et al.* IFN-α inhibits telomerase in human $CD8^+$ T cells by both hTERT downregulation and induction of p38 MAPK signaling. *J. Immunol. Baltim. Md 1950* **191**, 3744–3752 (2013).
13. Ng, C. T. & Oldstone, M. B. A. Infected CD8α- dendritic cells are the predominant source of IL-10 during establishment of persistent viral infection. *Proc. Natl. Acad. Sci. U. S. A.* **109**, 14116–14121 (2012).
14. Bazhin, A. V., von Ahn, K., Fritz, J., Werner, J. & Karakhanova, S. Interferon-α Up-Regulates the Expression of PD-L1 Molecules on Immune Cells Through STAT3 and p38 Signaling. *Front. Immunol.* **9**, 2129 (2018).

15. Kaser, A., Nagata, S. & Tilg, H. Interferon alpha augments activation-induced T cell death by upregulation of Fas (CD95/APO-1) and Fas ligand expression. *Cytokine* **11**, 736–743 (1999).

16. Fraietta, J. A. *et al.* Type I interferon upregulates Bak and contributes to T cell loss during human immunodeficiency virus (HIV) infection. *PLoS Pathog.* **9**, e1003658 (2013).

17. Herbeuval, J.-P. *et al.* Regulation of TNF-related apoptosis-inducing ligand on primary CD4+ T cells by HIV-1: role of type I IFN-producing plasmacytoid dendritic cells. *Proc. Natl. Acad. Sci. U. S. A.* **102**, 13974–13979 (2005).

18. Wilson, E. B. *et al.* Emergence of distinct multiarmed immunoregulatory antigen-presenting cells during persistent viral infection. *Cell Host Microbe* **11**, 481–491 (2012).

19. Swiecki, M. *et al.* Type I interferon negatively controls plasmacytoid dendritic cell numbers in vivo. *J. Exp. Med.* **208**, 2367–2374 (2011).

20. Kim, H.-J. *et al.* CD8 $^{+}$ T regulatory cells express the Ly49 Class I MHC receptor and are defective in autoimmune prone B6-Yaa mice. *Proc. Natl. Acad. Sci.* **108**, 2010–2015 (2011).

21. Anz, D. *et al.* Activation of melanoma differentiation-associated gene 5 causes rapid involution of the thymus. *J. Immunol. Baltim. Md 1950* **182**, 6044–6050 (2009).

22. Alvarez, M. D. L. *et al.* Cross-talk between IFN-alpha and TGF-beta1 signaling pathways in preneoplastic rat liver. *Growth Factors Chur Switz.* **27**, 1–11 (2009).

23. Aman, M. *et al.* Interferon-alpha stimulates production of interleukin-10 in activated CD4+ T cells and monocytes. *Blood* **87**, 4731–4736 (1996).

24. Le Buanec, H. *et al.* IFN- and CD46 stimulation are associated with active lupus and skew natural T regulatory cell differentiation to type 1 regulatory T (Tr1) cells. *Proc. Natl. Acad. Sci.* **108**, 18995–19000 (2011).

Supplementary Figure Legends

Supplementary Fig. 1: Gating strategy for immune cell types and specific markers analysed in each immune cell subsets.

(a) Gating strategy for immune cell types. The gating strategy used to identify the main cellular subsets is presented. Arrows are used to visualize the relationships across plots, and numbers are used to call attention to populations described here. After doublets and dead cells were excluded, lymphocytes were gated based on FSC-A/SSC-A properties. From the $CD14^{-}CD19^{-}$ lymphocyte gate, the following populations were identified: $CD3^{+}TCR\gamma\delta^{+}$, $TCR\gamma\delta^{-}$ were subdivided in $CD3^{-}$ and $CD3^{+}$T-cells. NK-cells were defined as CD3–TCRγδ–HLA-DR– and classified as early NK ($CD56^{+}$CD16–), mature NK ($CD56^{+}CD16^{+}$), and terminal NK (CD56–$CD16^{+}$) cells. The $CD3^{+}$TCRγδ– population was divided in $CD4^{+}$ and $CD8^{+}$ T-cells. In $CD4^{+}$T-cells subpopulation, $CCR7^{+}$ and $CD45RA^{+}$ were used to further classify these cells in four subpopulations: N ($CCR7^{+}CD45RA^{+}$), CM ($CCR7^{+}CD45RA^{-}$), EM ($CCR7^{-}CD45RA^{-}$) and TEMRA ($CCR7^{-}CD45RA^{+}$). Tregs were identified from the $CD4^{+}$ population using Foxp3 expression. $Foxp3^{+}$ cells were classified in naïve and memory Treg cells using CD45RA and CD25 markers. $CD45RA^{-}CD25^{+}$ represent the memory Treg cells population. As for $CD4^{+}$T-cells, $CD8^{+}$T-cells were classified using CD45RA and CCR7 markers: four populations were identified: N ($CCR7^{+}CD45RA^{+}$), CM ($CCR7^{+}CD45RA^{-}$), EM ($CCR7^{-}CD45RA^{-}$) and TEMRA ($CCR7^{-}CD45RA^{+}$). Among TEMRA $CD8^{+}$T-cells, we distinguished two cytotoxic subpopulations: $iKIR^{+}$ ($CD8^{+}$supp) and $iKIR^{-}$ (CTL). Dendritic cells (DCs) were identified by gating on $CD3^{-}CD19^{-}CD56^{-}CD14^{-}HLA^{-}DR^{+}$ and from there $CD123^{+}CD11c^{-}$ (pDCs) and $CD11c^{+}CD123^{-}$ mDCs were identified.

(b) Specific markers analysed in each immune cell subsets.

Supplementary Fig. 2: Immune cell types of non-ECs but not of ECs involved in the innate phase of an anti-HIV IR exhibit distinct altered pattern linked to elevated IFNα.

(a) Representative dot plot showing how to distinguish pDC ($CD123^{+}CD11c^{-}$) and mDC ($CD123^{-}CD11C^{+}$) subsets within the $lin^{-}HLA\text{-}DR^{+}$ population in HD **(aI).** Histograms showing the frequencies of pDC **(aII)** and the pDC:mDC ratio **(aIII)** across the groups (HDs n=22 (black), ECs n=12 (green) and non-ECs n=8 (red)). (**b**) Specific markers proportion on TCR γδ T-cells of each studied group (HDs n=22, ECs n=12 and non-ECs n=8)**.** (**c**) Histograms showing the frequencies of CCR7 in $CD4^{+}$ **(cI)** and $CD8^{+}$ **(cII)** T-cells across the groups (HDs n=22, ECs n=12 and non-ECs n=26). Scatterplots showing relationships between the frequencies of $CD4^{+}$CM **(cIII)** and $CD8^{+}$CM **(cIV)** T-cells with IFNα levels in HIV-1-infected patients. **(cV)** Histograms showing the frequencies of $CD8^{+}$CM in HDs (n=22), HLA-B57^{+} ECs (n=10), HLA-B57^{-} ECs (n=6) and non-ECs (n=26). Multiple group comparisons were assessed through Kruskal–Wallis test with Dunn's multiple comparison testing, and correlation with Spearman's rank correlation test. Graph show the median values and p values (*P<0.05, **P<0.01, ***P<0.001, ****P<0.0001). ns: not significative. Error bars on graphs represent interquartile ranges.

Supplementary Fig. 3: Frequency and phenotypic alterations of $CD4^{+}$T-cell subsets in non-ECs and ECs

Boxplots showing the expression of indicated marker in $CD4^{+}$Naïve (**a**), EM (**b**) and TEMRA (**c**) across the groups (HDs n=22 (black), ECs n=12 (green) and non-ECs n=8 (red)). (**d**) Scatterplots showing relationships between the expression level of indicated markers in the $CD4^{+}$CM subsets (ECs n=12 and non-ECs n=8). Multiple group comparisons were assessed through Kruskal–Wallis test with Dunn's multiple comparison testing, and correlation with Spearman's rank correlation test. Graph show the median values and p values (*P<0.05,**P<0.01, ***P<0.001, ****P<0.0001). ns: not significative.

Supplementary Fig. 4: Effect of IFNα and IFNλ2 on stimulated CD4+T-cells from HDs.

CD4+T-cells (1.5 × 105 per well) were stimulated with platebound anti-CD3 mAb (pbαCD3) (4 μg/mL) in presence of soluble anti-CD28 mAb (sαCD28) (4 μg/mL) and IL-2 (100 IU/mL) for 4 days. (aI) Representative FACS histograms displaying IFNα effect on CD4+T-cell proliferation measured by CFD dilution assay. (aII) Histograms showing dose–effect of IFNα and IFNλ2 on the frequency of CFDlow cells (n=4). (bI) Representative FACS histograms displaying IFNα effect on apoptosis of 4 d-stimulated CD4+T-cells evaluated by 7-amino-actinomycin D (7-AAD) staining. (bII) Histograms showing dose–effect of IFNα and IFNλ2 on the frequency of 7-AAD+ cells (n=4). (cI) Representative FACS histograms displaying IFNα effect on CD38 expression on 4 d-stimulated CD4+T-cells. Histograms showing the IFNα and IFNλ2 dose–effect on (cII) the CD38 frequency and (cIII) the CD38 Mean Fluorescence Intensity (MFI) in CD4+ T cells (n=4). (cIV) Representative FACS histograms displaying IFNα effect on CD25 expression on 4 d-stimulated CD4+T-cells.

Histograms showing the IFNα and IFNλ2 dose–effect on (cV) the CD25 frequency and (cVI) the CD25 MFI in CD4+T-cells (n=4). Multiple group comparisons were assessed through Kruskal–Wallis test with Dunn's multiple comparison testing. Graph show the median values and p values (*P<0.05, **P<0.01, ***P<0.001, ****P<0.0001). ns: not significative. Error bars on graphs represent interquartile ranges.

Supplementary Fig. 5: Frequency of CTL and CD8+supp in ECs-B57+, ECs-B57−, non-ECs and HDs.

Histograms showing distributions of CTL (a) and CD8+supp (b) between HDs (Black, n=24), ECs-B57+ (green, n=10), ECs-B57− (purple and blue, n=6) and non-ECs (red, n=26). One ECB57−(EC13) in blue behaves as an EC-B57+. Significance was determined by unpaired Mann-Whitney U test. *P<0.05, **P<0.01, ***P<0.001, ****P<0.0001. Multiple group comparisons were assessed through Kruskal–Wallis test with Dunn's multiple comparison testing. Graph show the median values and p values (*P<0.05, **P<0.01, ***P<0.001,

****P<0.0001). ns: not significative. Error bars on graphs represent interquartile ranges.

Supplementary Fig. 6: NK-cell cytotoxic activity varies in HIV-infected patients according to their status.

1-Left: in non-ECs, NK-cells are inactive given the high frequency of their inhibitorycheckpoints leading to a state of exhaustion in large part induced by IFNα.

2- Right and up: in HLA-B57+ ECs NK-cells, which possess negligible iKIR and further express negligible inhibitory receptors, lyse infected target cells expressing HLA-B restricted HIVpeptides.

3-Right and down: in HLA-B57- ECs NK-cells, which express iKIR, but have negligible inhibitory checkpoints, express the activating NKG2C receptor, counterbalancing the iKIR signaling, and can thereby kill infected cells carrying HIV-peptides in an HLA-E restriction.

Figure created with Biorender.

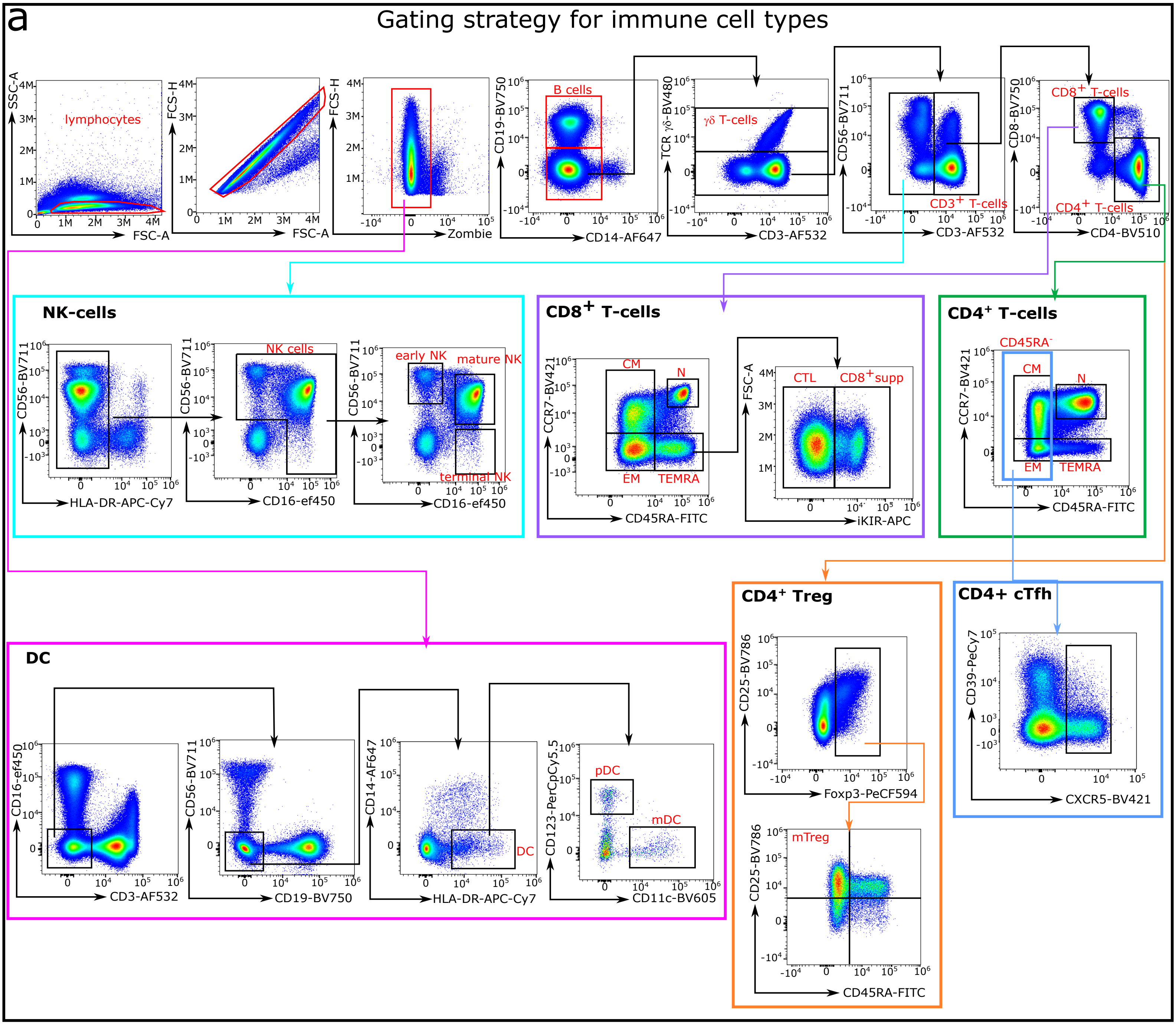

b

Specific markers analysed in each immune cell subsets

Immune activation / Maturation
CD25, CD28, HLA-DR,
CD26, CD39, CD38, CD95 and Helios

Immune checkpoint
PD1, CTLA-4, iKIR, NKG2A,
NKG2C, NCR, NKG2D

Immune cell function
GrzB/perf, Foxp3

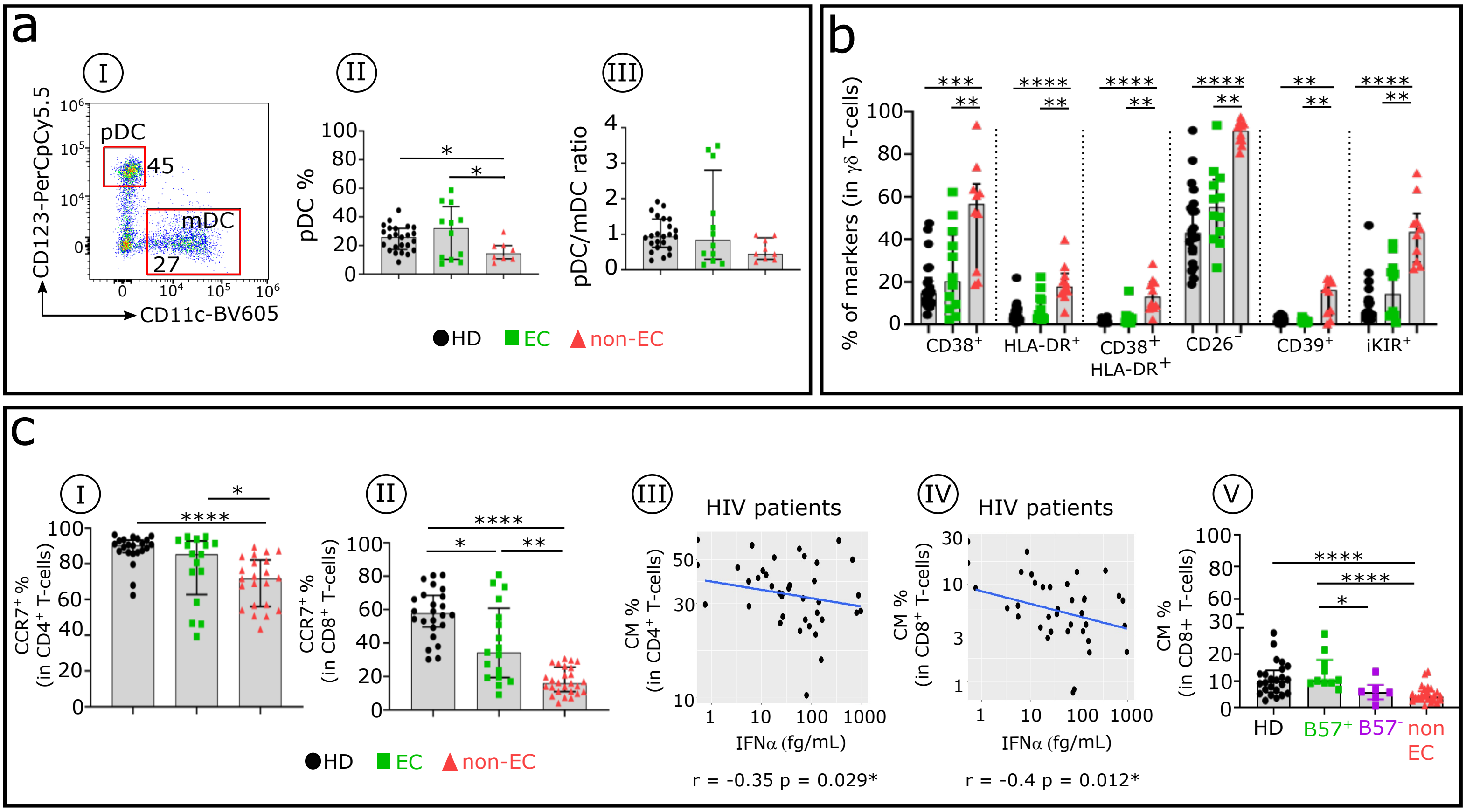
a
I
II
III
pDC
45
mDC
27
CD123-PerCpCy5.5
CD11c-BV605
pDC %
pDC/mDC ratio
HD EC non-EC
b
% of markers (in γδ T-cells)
CD38+
HLA-DR+
CD38+ HLA-DR+
CD26-
CD39+
iKIR+
c
I
II
III
IV
V
CCR7+ % (in CD4+ T-cells)
CCR7+ % (in CD8+ T-cells)
HIV patients
CM % (in CD4+ T-cells)
CM % (in CD8+ T-cells)
IFNα (fg/mL)
r = -0.35 p = 0.029*
r = -0.4 p = 0.012*
CM % (in CD8+ T-cells)
HD
B57+
B57-
non EC
HD EC non-EC

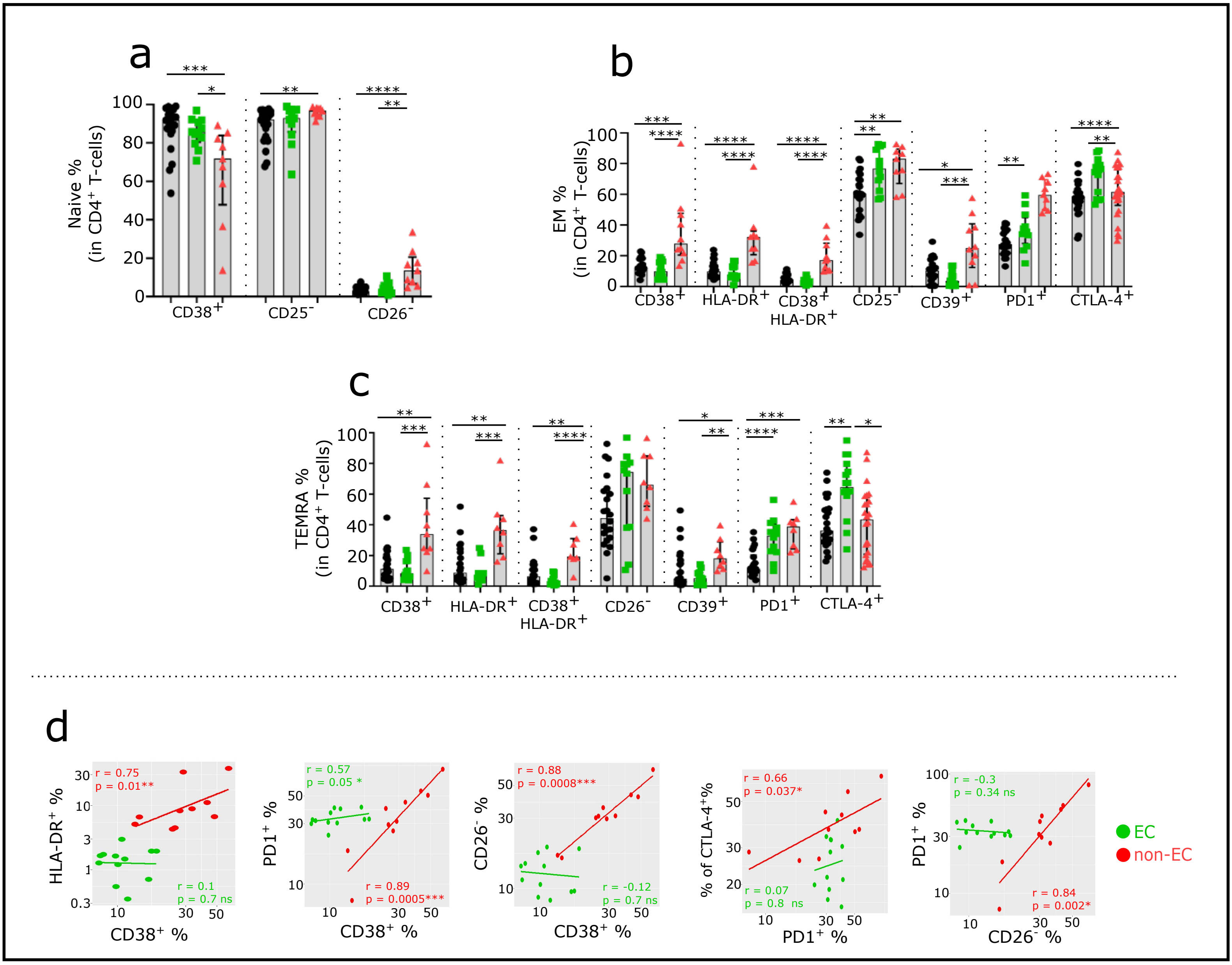

a
b
c
d
Naive %
(in CD4+ T-cells)
EM %
(in CD4+ T-cells)
TEMRA %
(in CD4+ T-cells)
CD38+
CD25-
CD26-
HLA-DR+
CD38+ HLA-DR+
CD39+
PD1+
CTLA-4+
HLA-DR+ %
PD1+ %
CD26- %
% of CTLA-4+%
CD38+ %
PD1+ %
CD26- %
r = 0.75
p = 0.01**
r = 0.1
p = 0.7 ns
r = 0.57
p = 0.05 *
r = 0.89
p = 0.0005***
r = 0.88
p = 0.0008***
r = -0.12
p = 0.7 ns
r = 0.66
p = 0.037*
r = 0.07
p = 0.8 ns
r = -0.3
p = 0.34 ns
r = 0.84
p = 0.002*
EC
non-EC

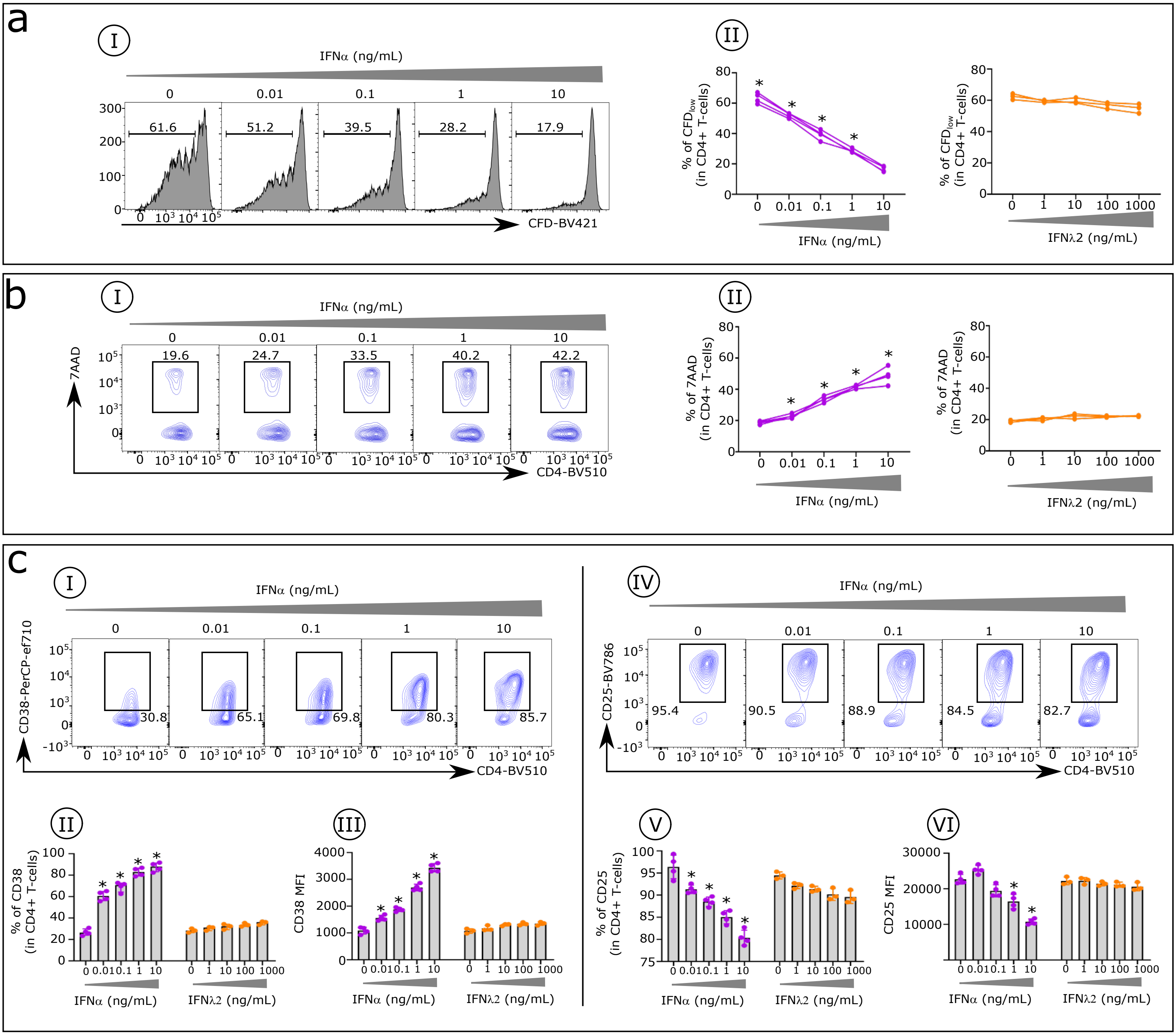

a
I
IFNα (ng/mL)
0
0.01
0.1
1
10
61.6
51.2
39.5
28.2
17.9
CFD-BV421
II
% of CFD low (in CD4+ T-cells)
IFNα (ng/mL)
IFNλ2 (ng/mL)
b
I
IFNα (ng/mL)
7AAD
19.6
24.7
33.5
40.2
42.2
CD4-BV510
II
% of 7AAD (in CD4+ T-cells)
IFNα (ng/mL)
IFNλ2 (ng/mL)
c
I
IFNα (ng/mL)
CD38-PerCP-ef710
30.8
65.1
69.8
80.3
85.7
CD4-BV510
IV
IFNα (ng/mL)
CD25-BV786
95.4
90.5
88.9
84.5
82.7
CD4-BV510
II
% of CD38 (in CD4+ T-cells)
III
CD38 MFI
V
% of CD25 (in CD4+ T-cells)
VI
CD25 MFI
IFNα (ng/mL)
IFNλ2 (ng/mL)

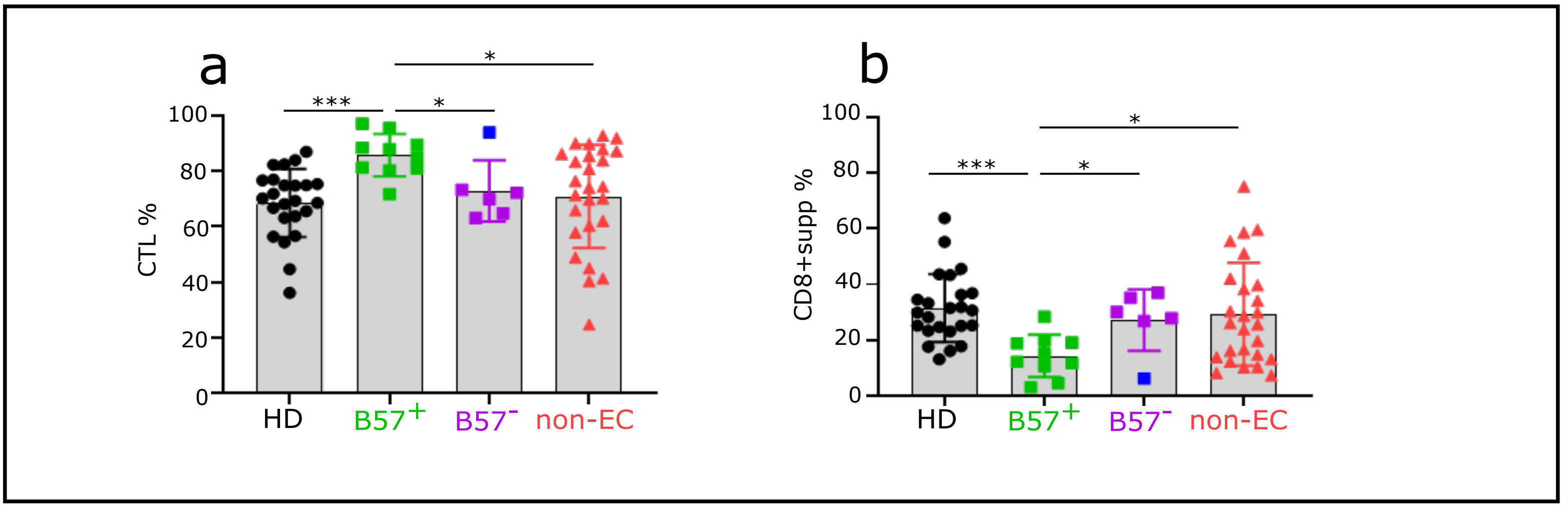

a
CTL %
100
80
60
40
20
0
***
*
*
HD
B57+
B57-
non-EC
b
CD8+supp %
100
80
60
40
20
0
***
*
*
HD
B57+
B57-
non-EC

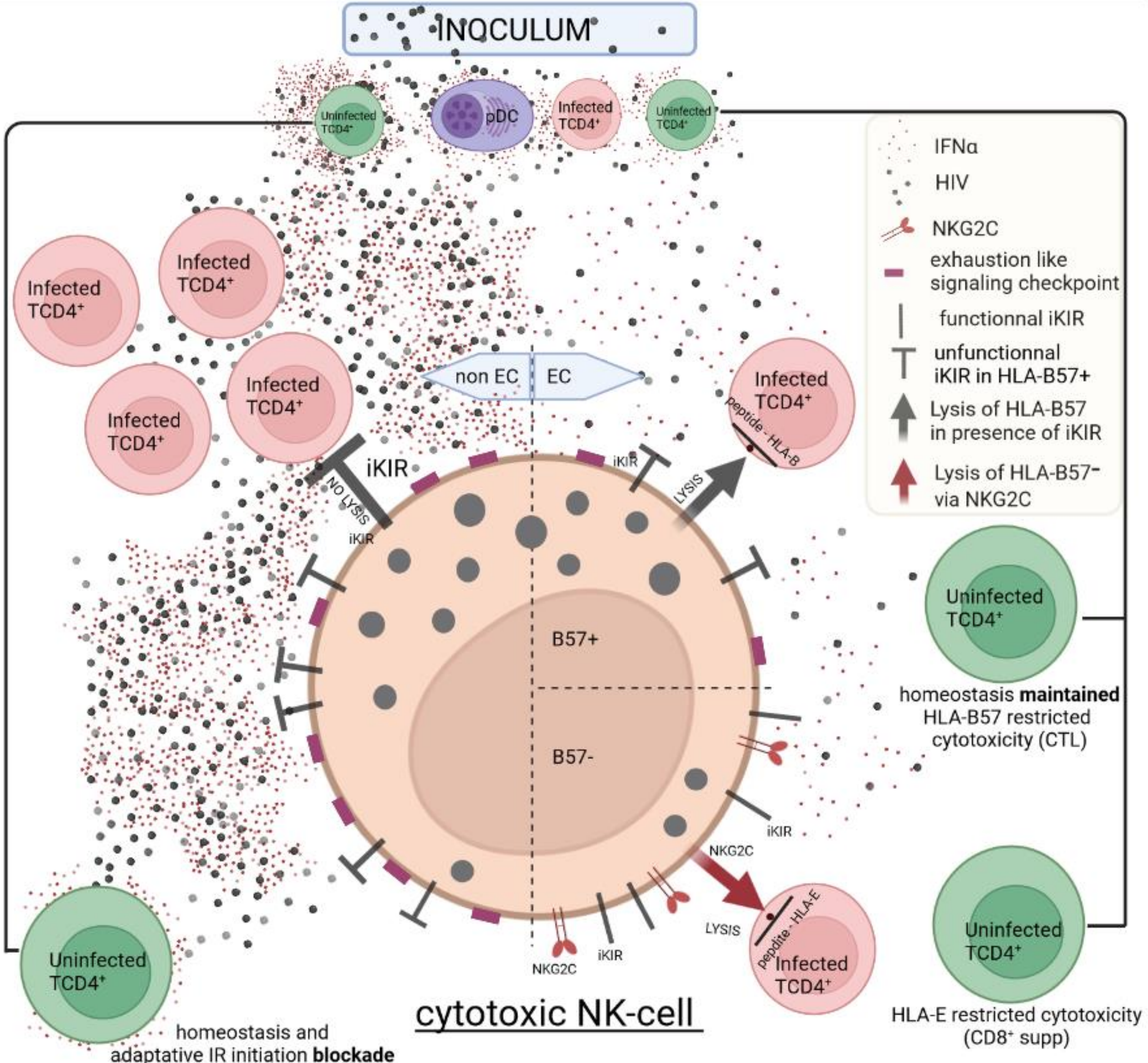
INOCULUM
Uninfected TCD4+
pDC
Infected TCD4+
Uninfected TCD4+
IFNα
HIV
NKG2C
exhaustion like signaling checkpoint
functionnal iKIR
unfunctionnal iKIR in HLA-B57+
Lysis of HLA-B57 in presence of iKIR
Lysis of HLA-B57- via NKG2C
Infected TCD4+
Infected TCD4+
Infected TCD4+
Infected TCD4+
non EC
EC
Infected TCD4+
peptide - HLA-B
iKIR
NO LYSIS
iKIR
iKIR
LYSIS
B57+
B57-
Uninfected TCD4+
homeostasis maintained
HLA-B57 restricted cytotoxicity (CTL)
iKIR
NKG2C
LYSIS
pepdite - HLA-E
Infected TCD4+
NKG2C
iKIR
Uninfected TCD4+
HLA-E restricted cytotoxicity (CD8+ supp)
Uninfected TCD4+
homeostasis and adaptative IR initiation blockade
cytotoxic NK-cell
