# Supplementary material for: Early elevated IFNα is a key mediator of HIV pathogenesis
Source: Commun Med (Lond). 2024 Mar 19;4:53. doi: 10.1038/s43856-024-00454-6 (PMC10951235; doi:10.1038/s43856-024-00454-6)
Supplement: Supplementary file 3 — Reporting Summary [file 43856_2024_454_MOESM3_ESM.pdf]

nature portfolio

Corresponding author(s): RC Gallo

Last updated by author(s): 2023/10/10

Reporting Summary

Nature Portfolio wishes to improve the reproducibility of the work that we publish. This form provides structure for consistency and transparency in reporting. For further information on Nature Portfolio policies, see our Editorial Policies and the Editorial Policy Checklist.

Statistics

For all statistical analyses, confirm that the following items are present in the figure legend, table legend, main text, or Methods section.

n/a	Confirmed	
☐	☒	The exact sample size (*n*) for each experimental group/condition, given as a discrete number and unit of measurement
☐	☒	A statement on whether measurements were taken from distinct samples or whether the same sample was measured repeatedly
☐	☒	The statistical test(s) used AND whether they are one- or two-sided<br>*Only common tests should be described solely by name; describe more complex techniques in the Methods section.*
☒	☐	A description of all covariates tested
☒	☐	A description of any assumptions or corrections, such as tests of normality and adjustment for multiple comparisons
☐	☒	A full description of the statistical parameters including central tendency (e.g. means) or other basic estimates (e.g. regression coefficient) AND variation (e.g. standard deviation) or associated estimates of uncertainty (e.g. confidence intervals)
☐	☒	For null hypothesis testing, the test statistic (e.g. *F*, *t*, *r*) with confidence intervals, effect sizes, degrees of freedom and *P* value noted<br>*Give P values as exact values whenever suitable.*
☒	☐	For Bayesian analysis, information on the choice of priors and Markov chain Monte Carlo settings
☒	☐	For hierarchical and complex designs, identification of the appropriate level for tests and full reporting of outcomes
☐	☒	Estimates of effect sizes (e.g. Cohen's *d*, Pearson's *r*), indicating how they were calculated

Our web collection on statistics for biologists contains articles on many of the points above.

Software and code

Policy information about availability of computer code

Data collection	FACS: patients immunophenotypes were performed using the five-laser (5L) Cytek Aurora (Cytek Biosciences) with SpectroFlo software (version 3.0.3). Cells cultures phenotypes were analysed using LSR Fortessa with FACSDiva software (Version 9.0)<br>Gene expression was performed using LightCycler 480 with LightCycler ® 480 Software (version 1.5)<br>serum cytokine level was analysed using SIMOA HD-1 Analyser (Quanterix) using the HD-1 Software 1.5.1606.30001
Data analysis	Flow Cytometry Standard (FCS) were analysed with FlowJo 10 (FlowJo LLC) and with cytobank<br>Gene expression was analysed with LightCycler 480 Software (version 1.5)<br>Data analysis was done with GraphPad Prism (Version 9.2.0) and with R:<br>R4.1.1 : https://www.r-project.org/<br>Rstudio 4.1.1 https://rstudio .com/<br>ggplot2 package https://ggplot2.tidyverse.org/reference/ggplot.html<br>Pheatmap package https://cran.r-project.org/web/packages/pheatmap/pheatmap.pdf<br>Dplyr https://cran.r-project.org/web/packages.dplyr/vignettes/dplyr.html<br>tidyr package : https://cran.r-project.org/web/packages/tidyr/index.html<br>ggforce package : https://cran.r-project.org/web/packages/ggforce/index.html<br>FactoMineR package : https://cran.r-project.org/web/packages/FactoMineR/<br>factoextra package : https://cran.r-project.org/web/packages/factoextra/index.html

For manuscripts utilizing custom algorithms or software that are central to the research but not yet described in published literature, software must be made available to editors and reviewers. We strongly encourage code deposition in a community repository (e.g. GitHub). See the Nature Portfolio guidelines for submitting code & software for further information.

Data

Policy information about availability of data

All manuscripts must include a data availability statement. This statement should provide the following information, where applicable:

- Accession codes, unique identifiers, or web links for publicly available datasets
- A description of any restrictions on data availability
- For clinical datasets or third party data, please ensure that the statement adheres to our policy

All data are available in the main Text, Figures, extended data Figures and Tables

Human research participants

Policy information about studies involving human research participants and Sex and Gender in Research.

Reporting on sex and gender	The clinical study protocol did not exclude or restrict enrollment based on sex or gender, rather than relied on other biological variables such as HIV infection status and ability to provide informed consent. Sex was collected by participant self-reporting and is provided in the manuscript.
Population characteristics	See above or in the manuscript.
Recruitment	Participants were recruited from primary care HIV clinical in the area, primarily by word of mouth and provider referral based on participant interest in volunteering in research studies. All patients signed informed consent.
Ethics oversight	The study was approved by University of Maryland, Baltimore IRB and by the ethical committee of Liège University hospital (full name: Comité d'Éthique Hospitalo-Facultaire Universitaire de Liège)

Note that full information on the approval of the study protocol must also be provided in the manuscript.

Field-specific reporting

Please select the one below that is the best fit for your research. If you are not sure, read the appropriate sections before making your selection.

☒ Life sciences ☐ Behavioural & social sciences ☐ Ecological, evolutionary & environmental sciences

For a reference copy of the document with all sections, see nature.com/documents/nr-reporting-summary-flat.pdf

Life sciences study design

All studies must disclose on these points even when the disclosure is negative.

Sample size	No formal sample size power calculation was performed. The number of subject in each experiments were indicated in each figure legend.
Data exclusions	No data were excluded.
Replication	The number of subject in each experiments were indicated in each figure legend.<br>For the analysis of cell phenotypes in culture, at least three independent assays was shown.
Randomization	no randomization was performed
Blinding	No blinding was necessary for our study

Reporting for specific materials, systems and methods

We require information from authors about some types of materials, experimental systems and methods used in many studies. Here, indicate whether each material, system or method listed is relevant to your study. If you are not sure if a list item applies to your research, read the appropriate section before selecting a response.

Materials & experimental systems

n/a	Involved in the study
☐	☒ Antibodies
☒	☐ Eukaryotic cell lines
☒	☐ Palaeontology and archaeology
☒	☐ Animals and other organisms
☒	☐ Clinical data
☒	☐ Dual use research of concern

Methods

n/a	Involved in the study
☒	☐ ChIP-seq
☐	☒ Flow cytometry
☒	☐ MRI-based neuroimaging

Antibodies

Antibodies used

CD3-AF532 (clone UCHT1 1/20 Invitrogen), CD4-BV510 (clone RPA-T4 1/40 Biolegend), CD8-BV750 (clone RPA-T8 1/300 Biolegend), CD56-BV711 (clone HCD56 1/150 Biolegend), CD56-APCCy7 (clone HCD56 1/80 Biolegend) CD16-ef450 (clone eBioCB163 1/600 Invitrogen), TCR-gd-BV480 (clone 11F2 1/40 BD Biosciences), CD19-BV750 (clone HIB19 1/40 Biolegend), CD14-AF647 (clone MOP9 1/300 BD Biosciences), CD123-PerCpCy5.5 (clone 7G3 1/10 BD pharmigen), CD11c-BV650 (clone 3.9 1/20 Biolegend), CD45RA-FITC (clone REA562 1/400 Miltenyi Biotech), CD45RA-BV421 (clone HI100 1/80 Biolegend), CCR7-BV421 (clone G043H7 1/40 Biolegend), CCR7-BV785 (clone G043H7 1/20 Biolegend) CD28-APC-R700 (clone CD28.2 1/80 BD Biosciences), CD25-BV786 (clone M-A251 1/40 BD Biosciences), HLA-DR-APCCy7 (clone 1243 1/1000 Biolegend), CD26-PE (clone BA5b 1/300 Biolegend), CD39-PeC7 (clone A1 1-300 Biolegend), CD38-PerCPeF710 (clone HB7 1/40 Invitrogen), PD1-BV650 (clone EH12.2H7 1/40 Biolegend), CTLA-4-PeCy5 (clone BNI3 1/20 BD Biosciences, KIR2DL1-APC (clone REA284 1/100 Miltenyi Biotech), KIR3DL1/DL2-APC (clone REA970 1/100 Miltenyi Biotech), KIR2DL2/DL3-APC (clone DX27 1-20 Miltenyi Biotech), KIR2DL5-APC (clone REA955 1/100 Miltenyi Biotech), Foxp3-PeCF594 (clone 236A/E7 1/100 BD Biosciences), zombie NIR (1/300 Biolegend), Helios-PE-Dazzle549 (clone 22F6 1/40 Biolegend), NKG2A-PE-Vio770 (clone REA110 1/400 Miltenyi Biotech, NKG2C-Viobright (clone REA205 1/100 Miltenyi Biotech), HLA-1a-BV711 (pentamer 1/20 Proimmune), HLA-E (clone E*01:01 1/20 Proimmune), GrzB/perf-PerCP-cy5.5 (clone QA16A02/B-D48 1/100 Biolegend), NKG2D-PE (clone AD11 1/20 BD Biosciences), CD95-BV421 (clone DX2 1/10 BD Biosciences), 7-AAD (1-50 Sigma Aldrich)

Validation

All antibodies are reactive against human and have been validated by the manufacturer by flow cytometric analysis

Flow Cytometry

Plots

Confirm that:

☒ The axis labels state the marker and fluorochrome used (e.g. CD4-FITC).

☒ The axis scales are clearly visible. Include numbers along axes only for bottom left plot of group (a 'group' is an analysis of identical markers).

☒ All plots are contour plots with outliers or pseudocolor plots.

☒ A numerical value for number of cells or percentage (with statistics) is provided.

Methodology

Sample preparation

For all studies, PBMCs were thawed and promptly washed. Subsequently, we proceeded with either antibody staining directly or in vitro activation with anti-CD3 and anti-CD28 abs in the presence or absence of cytokines and then antibody-based staining.

Instrument

Samples were acquired on a 5 laser Cytek Aurora or BD LSR Fortessa

Software

To collect the cells SpectroFlo software (version 3.0.3) or FACSDiva software (Version 9.0) were used.
Flow Cytometry Standard (FCS) were analysed with FlowJo 10 (FlowJo LLC) and with cytobank

Cell population abundance

No sorting experiments were conducted.

Gating strategy

Gating strategies are shown in the extended data. Briefly, all lymphocytes were initially gated using standard FCS/SSC gating, followed up by singlet discrimination. All subsequent manual gating was done with markers optimized to have clear distinction between positive and negative populations.

☒ Tick this box to confirm that a figure exemplifying the gating strategy is provided in the Supplementary Information.
